# Learning from the design and development of the NHS Safety Thermometer

**DOI:** 10.1093/intqhc/mzu043

**Published:** 2014-04-30

**Authors:** Maxine Power, Matthew Fogarty, John Madsen, Katherine Fenton, Kevin Stewart, Ailsa Brotherton, Katherine Cheema, Abigail Harrison, Lloyd Provost

**Affiliations:** 1 Haelo, Salford Royal NHS Foundation Trust, Stott Lane, Salford M6 8HD, UK; 2 Patient Safety Policy and Strategy, NHS Commissioning Board, 4–8 Maple Street, London W1 T 5HD, UK; 3 Health and Social Care Information Centre, 1 Trevelyan Square, Boar Lane, Leeds LS1 6EB, UK; 4 University College London Hospitals, 235 Euston Road, London NW1 2BU, UK; 5 Clinical Effectiveness & Evaluation Unit, Royal College of Physicians, 11 St Andrews Place, Regent's Park, London NW1 4LE, UK; 6 The Quality Observatory, York House, 18-20 Massetts Road, Horley, Surrey RH6 7DE, UK; 7 Associates in Process Improvement, Austin TX, USA

**Keywords:** harm, measurement, testing

## Abstract

**Quality issue:**

Research indicates that 10% of patients are harmed by healthcare but data that can be used in real time to improve safety are not routinely available.

**Initial assessment:**

We identified the need for a prospective safety measurement system that healthcare professionals can use to improve safety locally, regionally and nationally.

**Choice of solution:**

We designed, developed and implemented a national tool, named the NHS Safety Thermometer (NHS ST) with the goal of measuring the prevalence of harm from pressure ulcers, falls, urinary tract infection in patients with catheters and venous thromboembolism on one day each month for all NHS patients.

**Implementation:**

The NHS ST survey instrument was developed in a learning collaborative involving 161 organizations (e.g. hospitals and other delivery organizations) using a Plan, Do, Study, Act method.

**Evaluation:**

Testing of operational definitions, technical capability and use were conducted and feedback systems were established by site coordinators in each participating organization. During the 17-month pilot, site coordinators reported a total of 73 651 patient entries.

**Lessons learned:**

It is feasible to obtain national data through standardized reporting by site coordinators at the point of care. Some caution is required in interpreting data and work is required locally to ensure data collection systems are robust and data collectors were trained. Sampling is an important strategy to optimize efficiency and reduce the burden of measurement.

## Quality issue

Research indicates that ∼10% of patients are harmed by healthcare [1, 2]. In these cases, patients and families often report a negative experience and adverse effects on psychological and social well-being [3, 4]. Reported figures on the burden of harm are mostly based on extrapolations, reported events, research or incomplete data fraught with methodological limitations [5–7]. Consequently, our understanding of the aggregate impact of harm at the national level is significantly impaired by a lack of robust data.

Harm occurs in all healthcare settings but occurrences are hard to measure, particularly outside hospital. Traditional efforts to detect harm have focused on voluntary reporting and tracking of ‘adverse incidents’. Alternative measurement approaches rely on administrative data, reviews of medical records using trigger tools and local audits [8, 9]. Measurement of safety outcomes is therefore largely retrospective, which is important for learning, but challenging for improvement. Improvement requires actionable ‘real-time’ data which engages, educates and mobilizes frontline staff to make changes at the point of care. Arguably, a more effective method of collecting data on harm is via prospective surveillance at the point of care but this can be dismissed as too expensive or difficult to implement.

## Initial assessment

In 2010, the Department of Health in England commissioned the Quality, Innovation, Productivity and Prevention (QIPP) programme comprising 12 domains (known as national work streams) and government policy moved to focus on improving outcomes. The QIPP safety work stream focused on four high volume harms (safety outcomes), pressure ulcers, falls, urinary tract infection (UTI) in patients with catheters and venous thromboembolism (VTE)—two of which (pressure ulcers and VTE) were highlighted as improvement areas in Domain 5 (safety) of the NHS Outcomes Framework [10].

Collectively, these harms were estimated to affect over 200 000 patients per year and cost £430 million in England alone [11]. An improvement collaborative, ‘Safety Express’, was planned to reduce the four harms; however, despite inclusion of these harms in the Outcomes Framework, reliable data were not available.

## Choice of solution

Our aim was to set up a low-cost pragmatic system to provide monthly data on four harms across care settings and produce measures that could be used locally for improvement but also aggregated to determine the burden of harm nationally. To accomplish this, during July 2010 to December 2011 we designed, developed and implemented a tool: the NHS Safety Thermometer (NHS ST). The instrument was not intended to be a comprehensive measure of harm but to provide a ‘temperature check’—hence the term ‘thermometer’—and was to be used alongside local measurement systems.

## Design principles

Design principles for the instrument were agreed by the development group, as follows:

- *Clinically valid* with clear operational definitions for harm *outcomes* (in this case, pressure ulcers, falls, UTI in patients with catheters and VTE).
- *Efficient*: it should not take >10 min per patient and must fit within the daily work flow of frontline clinicians.
- *Equitable* and capable of being used wherever the patient is located (e.g. in a home, community or hospital setting).
- *Timely*: giving an immediate summary of results that can be used by teams in their improvement work.
- *Patient focused*: measuring the absence of all four outcomes in individual patients ‘harm free’ care as well as the individual harms.
- *Focused on all harm* irrespective of perceived avoidability or attribution.
- *Easy to aggregate* to show results at the ward, organization, region or national levels.

## Approach to implementation

**Figure 1:**
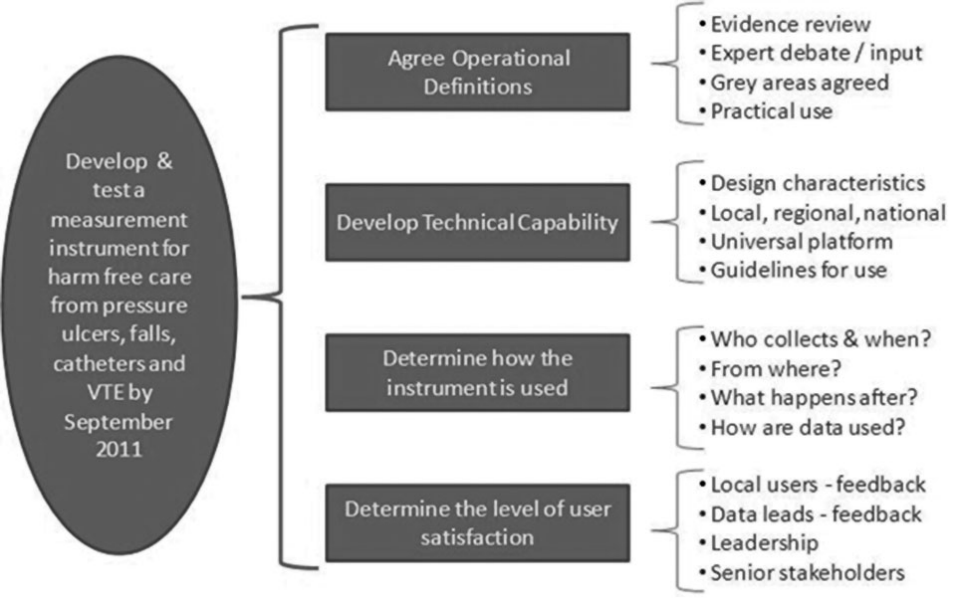
*Framework of project plan*: a series of tests were conducted within each of the primary portfolios of work to develop and refine operational definitions; develop technical aspects of the instrument; study its use, including the organization of data collection, sampling strategy, accessing data sources and interpretation. On-going tests focused on the appeal of the instrument to frontline teams.

A plan for developing the instrument was constructed using the Project Plan Framework seen in Fig. 1. The testing was segmented around four portfolios of work:

- Agreeing on the operational definitions.
- Technical development of the spread sheet-based collection instrument.
- Guidance for instrument use and data collection.
- Feedback and satisfaction with the instrument.

### Agreeing on the operational definitions

Specialist expert groups were commissioned for each specialty area. These groups met monthly during the first 6 months of the development. They were asked to review the literature and recommend operational definitions to the steering group for testing based on consensus.

### Technical development of the spread sheet-based collection instrument

In July 2010, the paper-based prototype instrument developed by the development group was tested by a small cohort of 12 testers who were all senior nurses employed in four pilot sites. Amendments were made after these four Plan, Do, Study, Act (PDSA) cycles based on responses from these organizations, and a second version was distributed with clearer instructions. This cycle of test and learn was repeated four times, increasing the number of test wards to four by version four of the paper-based instrument. During cycle five of testing, the form was transferred from a word document to an Excel workbook. Following feedback, drop-down lists were added to the Excel sheet for easier selection of choices and definitions. Visual Basic code was included to save survey records in a standard format.

The next stage of the process was the creation of a ‘merge’ function; this allowed an organization to merge surveys from its pilot wards in one step, and to merge surveys across organizations. At the same time, charting functions were added to the evolving NHS ST application so that frontline teams could view and print time series charts as soon as they had inputted their data.

### Guidance for instrument use and data collection

Organizations were told to test on the same four units each month, but some also experimented in different settings (specialty units, community and patients home). During testing, one region, NHS South East Coast (SEC), mandated the use of the instrument quarterly in acute care, resulting in a natural experiment in which a population of 4.2 million (26 providers) surveyed 50% of hospitalized patients once per quarter. We used these data as a comparator group to determine the validity of the national data.

The instrument was designed to be used by frontline clinical teams during a conversation with the patient, data were collected on 100% of patients in NHS care whether or not they were perceived to be at risk of harm. For every patient, the clinician collects information on age band (<18, 18–70, 70+), gender (male/female), location (hospital ward, community hospital, hospice, nursing home, own home and residential care home), specialty and harm profile. Specifically, the clinicians would examine the patient, review the notes and speak to the patient to determine the presence of pressure ulcers, falls, UTI in patients with a urinary catheter, the management of VTE risk and the presence of a VTE. Data for each patient were recorded on a standardized pro-forma by the clinical team. For each clinical unit, a master form would be submitted by the local leader to the site coordinator who would collate the site level data to submit to the national team [12].

### Feedback and satisfaction with the instrument

Systems were established to gain feedback from the field tests, reporting into the development group. Principally, these feedback mechanisms were from the following:

- Interaction with testers (site coordinators), which was organized through fortnightly on line meetings.
- A web forum was hosted on the ‘Patient Safety First’ platform and used to respond to email queries [13].
- Regional leads in the 10 Strategic Health Authorities fed back from frontline teams and measurement leads in each of the organizations in their regions.
- Measurement surgeries for frontline teams at Safety Express learning events were held at 9 of 12 face-to-face meetings (open to over 1000 attendees).
- Bespoke regional Measurement Workshops were organized by 6 of the 10 SHA leads to build measurement for improvement capabilities and to share learning.

### Measuring professional satisfaction

A questionnaire survey was carried out on data collection day in September 2010 to collect feedback from users. For this questionnaire, out of nine questions asked, four questions used a five-point categorical scale to determine an average rating. Using this scale, the most positive responses would be scored 5. For example, when describing the importance of the safety outcomes from a user perspective, a rating of 5 was given to the response ‘strongly agree’, 4 to ‘agree’, 3 to ‘undecided’, 2 to ‘disagree’ and 1 to ‘strongly disagree’. Using these scores, we calculated a rating average by adding up the weighted responses (numerator) and dividing by the overall number of responses (denominator). A summary of results from the five-point categorical scale survey questions are shown in Table 1.

**Table 1:** Details of questions and responses from users of the NHS Safety Thermometer Survey from 2010

Questions	Answer options*	Rating
The four safety outcomes identified in the safety thermometer are important for our patients	Pressure ulcers	4.05
	Urine infections	3.78
	Catheter use	3.84
	Harm from falls	3.90
Response options: *1 (strongly disagree) to 5 (strongly agree)*	VTE	3.96
How useful was the support you received?	From regional leads	3.82
	From PSF website	3.20
	From Web Ex	3.20
	From slides	3.39
Response Options: *1 (not helpful at all) to 5 (very helpful)*	From fact sheet	3.09
What did you learn the most about?	Pressure ulcers	3.25
	Urine infections	3.21
	Catheter use	3.28
	Harms from falls	3.18
Response options: *1 (nothing) to 5 (a lot)*	VTE	3.54
Which clinical areas are you likely to include in your next test?	Orthopaedics	4.00
	Medical	4.27
	Rehabilitation	3.80
	Surgical	3.95
	Community	3.62
	Mental health	2.64
	Paediatric	3.28
	Nursing home	2.86
Response options: *1 (highly unlikely) to 5 (highly likely)*	Other	3.18

*For each question, five response options were given and a five-point scale was used to determine agreement or satisfaction.

### Data analysis

Statistical analysis was performed using R-2.15.1 for Windows [14]. Run charts and p-charts were produced in Excel. Data are presented as count or proportions. Exact binomial confidence intervals (CIs) were calculated for proportions. Statistical ‘significance’ was predefined at the 5% level. *χ*^2^ tests were used to assess the relationship between proportions.

### Ethical issues

No patient identifiable data were collected. The data were collected as part of the routine pattern of care and the burden on the patient was evaluated throughout and found to be minimal. Completed NHS ST templates were stored on password-protected NHS computer systems or devices.

## PDSA testing and instrument refinement

One hundred and sixty-one organizations participated, with a total of 73 651 patients. Ninety per cent of data were submitted from hospital settings, 3% from the patients' own home, 2% from nursing homes and 5% from other settings. Over 50% of the wards settings chosen by participants for testing in hospitals were medical. Seventy-one per cent of organizational monthly submissions contained at least 30 patients and 84% achieved at least 20 patients.

Testing and refinement of the NHS ST involved the PDSA method [15]. This methodology enhances the chances of application at scale as it tests a planned change in a ‘live’ setting and considers its strengths and weaknesses before adapting it for further testing.

### Agreeing on the operational definitions

Multiple iterations of tests were performed in the development of the definitions. Tables 2–5 give a summary of learning and feedback, which shaped the final definitions. Data produced by the NHS ST indicated that 7.4% patients had a pressure ulcer (categories 2–4), 17% of patients had an in-dwelling urinary catheter, 2% of patients had a catheter and were also being treated clinically for a UTI, 1.5% of patients were being treated clinically for a new VTE and 1.3% of patients had a fall resulting in harm. Data from the South East Coast SHA were not significantly different from the national data (Table 6).

**Table 2:** Development process for the operational definition of pressure ulcers

Plan	Use European Pressure Ulcer Advisory Group Guidelines for definition and classification of the pressure ulcers (PU) [18]. Collect data from clinical records, inspection of skin and document the percentage of patients with a pressure ulcer on the day of survey. Limit the time for data collection to <2 min and ensure the measure is applicable in all healthcare settings		
Do	Carry out the test and gain feedback from frontline teams and measurement experts over repeated tests using feedback from on-line forums, worksheets and verbal report. Share a summary of this information with tissue viability expert groups immediately (after the first tests and then quarterly)		
Study	There were challenges with the use of classification scales, in particular the skills of frontline nurses to apply them reliably (most confusion came with recognizing the difference between category II pressure ulcers and moisture lesions) and the time taken to collect the information exceeded the 2 min maximum (imposed by the design principles) where multiple PUs were being recorded and categorized. System leaders wanted a measure which would determine whether the PU was new or old		
Act	Guidance was added to the instrument giving a clear steer on the pressure ulcer grading system. Education and training materials were developed including picture libraries to aid classification. It was agreed to collect data on the patient's worst pressure ulcer, instead of all. An ‘old and new’ category was added on advice from experts (based on a lead time of 72 h for a pressure ulcer resulting from deep tissue injury) [18]		
Unresolved issues	Contention remains about the 72-h window to determine whether a pressure ulcer is ‘old’ or ‘new’ based on content knowledge about the time frame for development of pressure ulcers (which are known to occur within hours if management is suboptimal) Data quality is contingent on the skills of frontline teams to apply the classification scales and continuous training and monitoring is required or alternative confirmatory opinions are required Not all pressure ulcers are captured and confusion exists between the new measure collected here (which gives a measure of ‘new’ occurrences) and an incidence rate		
Final definition	P1	P2	P3
	The proportion of patients with an old pressure ulcer (present on admission to the organization, or developed within 72 h) documented following a skin inspection	The proportion of patients with a new pressure ulcer (not present on admission to the organization and developed after 72 h) documented following a skin inspection	The proportion of patients with any (new or old) pressure ulcers documented following skin inspection on the day of the survey
	Each measure can be viewed by categories (2–4)

**Table 3:** Development process for the operational definition of falls

Plan	Use the National Patient Safety Agency definition for the classification of falls and the severity of harm from falls [19]. Collect data from clinical records and a conversation with the patients on whether the patient has fallen in the last 24 h. Limit the time for data collection to <2 min	
Do	Carry out the test and gain feedback from frontline teams and measurement experts over multiple cycles of testing using feedback from on-line forums, worksheets, feedback at face-to-face meetings and verbal report. Share a summary of this information with falls experts immediately (after the first tests and then quarterly)	
Study	Testing demonstrated that clinical teams were unhappy with the time frame (of 24 h) originally recommended by the steering group. The time limit was used to ensure compliance with the design principle of efficiency; however, in practice, teams found that patients had fallen and experienced harm but were being missed during data collection. Testing demonstrated that clinicians wanted to clarify about the location of the fall, for example did a fall in the street count? There were a significant number of patients surveyed with harm from falls being missed because the 24-h window was too narrow. Varying interpretations of the harm classifications in particular around the distinction between ‘no harm’ and low harm. Anecdotal evidence of patients having said ‘yes’ when asked if they'd fallen, with staff having not known or unclear information in patient notes. Positive feedback suggested staff feel reviewing falls is an opportunity to interact with patients and highlight the importance of patient safety	
Act	Guidance was added to the instrument to indicate that users were to document only those falls that happened in a care setting in the previous 72 h. The review time for 72 h was tested and found that it was possible to review 72 h of case notes within the 2-min allocation. Guidance was provided on the use of the harm allocation and advised that the harm was physical rather than psychological (whilst recognizing the importance of the fear of falling)	
Unresolved issues	Contention remains over the inability of the instrument to record the total burden of harm from falls (i.e. a count of all falls, not simply those that happened in the last 3 days) Content experts are not yet agreed that this measure adds value when compared with incident reports or that data from a point estimate offer additional value	
Final definition	F1	F2
	The proportion of patients with evidence of a fall in a care setting in the last 72 h (including home if on a DN caseload), from discussion with the patient and review of clinical notes reviewed on the day of the survey.	The proportion of patients with evidence of harm from a fall in a care setting in the last 72 h (including home if on a DN caseload), from discussion with the patient and reviewed on the day of the survey.
		This measure can be viewed by harm severity.

**Table 4:** Development process for the operational definition of catheter-associated UTI

Plan	Use the agreed definition for catheter-associated UTI developed by the Health Protection Agency for patients with an in-dwelling urinary catheter. When trying to agree a definition it became clear that the association between catheters and UTI was complex requiring laboratory tests and microbiology expertise [20]. Therefore, two proxy measures were used:		
	Does this patient have an in-dwelling urinary catheter? Are they being treated clinically for a urinary tract infection?		
	Limit the time for data collection to <2 min		
Do	Carry out the test and gain feedback from frontline teams in all settings, using feedback from on-line forums, worksheets, feedback at face-to-face meetings and verbal report. Share a summary of this information with experts immediately (after the first tests and then quarterly)		
Study	Testing revealed that clarification was required about the inclusion/exclusion criteria, e.g. why were supra-pubic catheters not included. Advice was required for those patients being trialled without catheter (but for whom there was still a risk of infection, having had a catheter *in situ* in the last 72 h). ‘Treatment’ was clarified as treatment with antibiotics and the symptoms of UTI were specified. (subsequent tests led to removal of the clinical symptoms, which were replaced with ‘local guidance’)		
Act	Questions were amended to ask whether the patient has, in the last 72 h, had an in-dwelling urinary catheter *in situ*. It was reaffirmed that the catheters to be documented were in-dwelling urinary catheters and gave an exclusion list in the instrument. The urinary infection question was changed to ‘is this person being treated with an antibiotic for a clinically diagnosed urine infection?’		
Unresolved Issues	The instrument is still unable to measure catheter-associated UTI and is reliant upon users understanding that the measure is a composite of two measures: the treatment of UTI in patients and the presence of an in-dwelling urinary catheter The margins of avoidability are contentious		
Final definitions	C1	C2	C3
	The proportion of patients with an in-dwelling urethral urinary catheter present on the day of survey or removed in the last 72 h	The proportion of patients with an in-dwelling urethral urinary catheter also receiving treatment for any UTI (on the basis of notes, clinical judgement and patient feedback)	The proportion of patients with an in-dwelling urethral urinary catheter also receiving treatment for a new UTI (on the basis of notes, clinical judgement and patient feedback)
		This measure can also be viewed by old UTI The proportion of patients (without catheters) being treated for UTI can also be viewed

**Table 5:** Development process for the operational definition of VTE

Plan	Use the definition recommended by the National VTE board for VTE (the collective name for pulmonary embolism and deep vein thrombosis). This definition was clinically complex, requiring a high level of training and testing and proved impossible to agree in the time available given the design limitations. A proxy measure was used: ‘is this patient being treated with anticoagulants for a clinically diagnosed VTE episode?’ Limit the time for data collection to >2 min		
Do	Carry out the test and gain feedback from frontline teams in all settings, using feedback from on-line forums, submitted worksheets, feedback at face-to-face meetings and verbal report. Share a summary of this information with VTE experts immediately (after the first tests and then quarterly)		
Study	There were challenges for frontline nurses in determining the response to this question:		
	There was confusion with the fact that anticoagulants can be used both prophylactically to prevent VTE and, clinically, to treat VTE A number of patients surveyed were on longstanding anticoagulation for long-term VTE management and it was unclear how data on these patients would be documented and used Experts were concerned that patients were documented as having a new VTE when it could be medically unavoidable. Conversely, even hospital acquired VTE events occur remote from the index hospitalization resulting in readmission to another division or hospital Use of the VTE indicator outside acute care was very difficult because of the limited information in records in patient's homes		
Act	It was agreed to expand the number of measures in the instrument for VTE and separate VTE into three logical steps:		
	‘Has the patient received a risk assessment?’ If at risk, has the patient received prophylaxis according to NICE guidance Is the person being treated for a VTE? In each case the drop down allowed the user to enter ‘not applicable’		
	The measures would be used only for hypothesis generating and learning and measures from settings outside hospital would not be published in external reports		
Unresolved issues	The specialist VTE community continues to be sceptical about the VTE outcome measure. Their argument is based on the research evidence which demonstrates that whilst post-surgical VTE episodes are largely avoidable, a significant number of VTE events are medically unavoidable and therefore calling these VTE events ‘harms’ is misleading and may have unintended consequences VTE is a condition that may be prevented in one setting but may occur (as a new admission) in another. For example, if post-surgical VTE prophylaxis is mismanaged, the patient may be discharged from one setting but re-present in another with new symptoms of VTE associated with the previous surgery. This suggests that one organization is potentially counting the harm attributable to another The changes observed through testing with this measure have, predominantly, been through the involvement of frontline nursing staff. This has both advantages and disadvantages. The training requirements for nursing staff to complete this measure accurately should not be underestimated		
Final Definition	V1	V2	V3
	The proportion of patients with a documented VTE risk assessment	The proportion of ‘at-risk’ patients receiving appropriate prophylaxis (in accordance with local guidance)	The proportion of patients receiving prescribed anticoagulation treatment (heparin, warfarin or equivalent) for treatment of a clinically documented VTE event
			Each measure can be viewed by category (DVT/PE/Other) This measure can be viewed by old and new VTE
	V1 and V2 were based on NICE guidance [21]

**Table 6:** Comparison of proportions for each measure (upper and lower 95% CI) using a chi-square goodness-of-fit test found no difference between the groups

Measure	National (*n* = 73,651) (proportion and 95% confidence interval)	South East Coast (*n* = 7130) (proportion and 95% confidence interval)	*χ*^2^	Significance *P*
P1: old PUs	6.5 [CI 3.04, 13.64]	6.73 [CI 3.04, 12.17]	0.002	0.99
P2: new PUs	1.02 [CI 0.61, 5.61)	1.16 [CI 0.25, 6.45]		
P3: all PUs	7.36 [CI 3.64, 14.9]	7.8 [CI 3.77, 13.17]		
F1: all falls	3.22 [1.12, 9.04]	2.97 [0.64, 7.72]	0.001	0.99
F2: falls with harm	1.26 [0.27, 6.1]	1.17 [0.24, 6.45]		
C1: catheterization	16.73 [10.43, 26.54]	14.15 [9.83 19.23]	0.322	0.85
C2: any UTI with catheter	2.03 [0.54, 7.22]	2.72 [0.64, 7.73]		
C3: new UTI with catheter	1.04 [0.16, 5.62]	1.44 [0.25, 6.4]		
V1: VTE risk assessment	69.27 [55.48, 85.92]	61.96 [42.73, 83.6]	1.441	0.69
V2: VTE prophylaxis	55.16 [42.91, 70.51]	59.06 [38.5, 80.3]		
V3: new VTE	1.51 [0.32, 6.42]	3.27 [1.14, 8.92]		
V4: old VTE	2.13 [0.59, 7.38]	3.23 [1.14, 8.92]		

### Technical specification of the spread sheet-based data collection instrument

**Figure 2:**
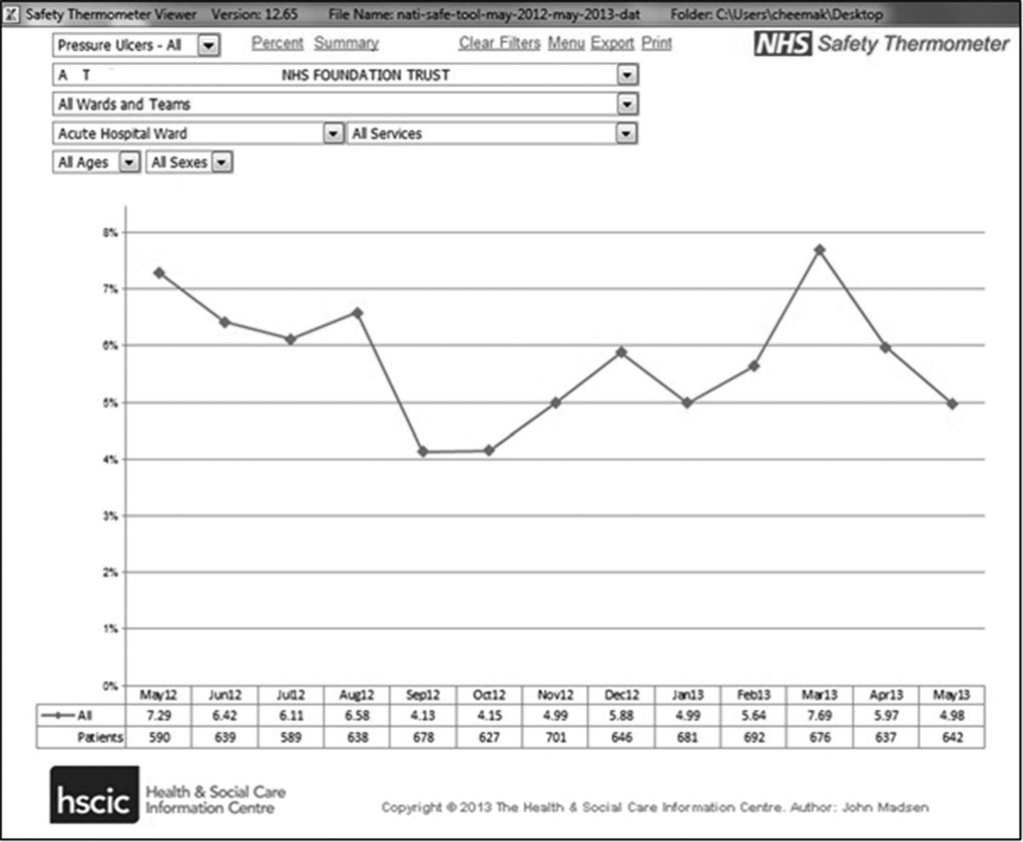
*Graphical display:* the data display sites that can provide information relating to the data they have inputted. The display visually shows progress over time in relation to the four harms. It can also provide the opportunity to gain more granular information if required.

The final NHS ST is self-contained with no requirements for a network connection and is an Excel Visual Basic application of MS Excel (Excel 97-2003 onwards). The same application is used for frontline data collection, charting and reporting. The interface is designed to validate data and minimize burden through a combination of drop-down lists and cross-validation checks. Feedback is provided to frontline users and includes four data views: a survey form, a summary dashboard, time series charts (Fig. 2) and a comparison report. It provides a function to merge data allowing surveys from any number of STs to be merged into a single new ST. The same application may be used by wards, teams, organizations and at a national level. Guidance is given in the instrument itself and in companion documentation to address common questions which arose from the testing [16].

### Observations of use and recommendations for data collection

Between September 2010 and December 2011, site coordinators used the instrument to report data. These were typically nurses, with support from nurse specialists, junior doctors and informatics. Patient voice was fed back through teams completing the testing. Patients were not directly asked for feedback. Learning from these tests and the technical training were shared on a web forum. The cycles of testing and learning continued as we received feedback and continued to develop the Excel-based tool. Each new version of the NHS ST (with updates from testing) was modified and distributed by email to the site coordinators. Each version was designed to be compatible with older versions to ensure data collection in earlier versions was preserved [17]. In total, there were 20 versions of the NHS ST.

The final sample size to be reported by each organization was calculated by determining the number of surveys required for a lower control limit on a proportions chart (p-chart) for each outcome. The highest value across the four types of harms was selected to determine the number of patients surveyed overall. Data from the first 6 months of testing (Table 7) were used to determine the sample size required to produce lower control limits ranged from 43 (for catheters) to 625 (for harm from falls). The steering group were advised that a sampling strategy of 50% of patients would not result in useful charts at a local level. It was concluded that 100% of patients should be surveyed to ensure all outcomes had robust charts for interpretation [22].

**Table 7:** Calculations of the number (*n*) of patients required to provide workable upper and lower control limits (LCL) on a proportions chart (p-chart) for each measure

Average:	Pressure Ulcers			Falls		Catheters	Catheter	VTE	
	New	Old	All	All	Harm		w. UTI	New	Old
**Average**	2.12	6.16	8.28	3.47	1.43	17.64	2.5	1.98	2.32
**n for p chart**	142	49	37	87	210	18	120	152	130
**n for LCL on chart**	418	138	100	252	625	43	353	448	381

### Satisfaction with the instrument

Sixty-three participants responded to the Questionnaire Survey in September 2010; 49% of respondents worked in the South, 25% in the Midlands and 25% in the North. Seventy-one per cent of respondents were from hospitals, 22% community and the remaining from nursing homes, specialist providers (mental health, children's and ambulance) and home nursing. Seventy-seven per cent were able to complete the survey in <15 min per patient, and the remainder (23%) stated that it took them, on average, 16–25 min. Forty-seven of the respondents completed the free-text box asking what they planned to do with the data. Example suggestions included: ‘Use the survey to instigate change in practice’, ‘Survey results will form the basis of action plans’, ‘Try to do more vigorous assessments on VTE’, ‘Use as a baseline and compare with other units to find best practice areas’ and ‘Share the data across the organization and discuss how the sample size and data collected might be more useful from the community setting’. Results from Question 8 illustrated that, when asked: ‘Would you participate in the Safety Thermometer survey again’, 85% of users confirmed they would, inferring some degree of satisfaction with the survey.

## Lessons learned

### It is possible to develop a system for measuring harm nationally through standardization and merging of locally reported data

The data collated have value at a national level in determining, for the first time, burden of harm from the four identified outcomes over time. A primary objective of this programme was to develop an instrument which could be used to track outcomes over time to determine the impact of the ‘Safety Express’ national programme. We reviewed the SEC data and compared it with the national data to determine whether the sample of four wards (used in the national data and aggregated to produce the final measures) produced skewed estimates when compared with the whole population of patients in NHS care in the SEC region. No significant difference was found between the samples (Table 6).

We also wanted to ensure measures had face validity with clinicians and produced data which were concordant with epidemiological studies. Research suggests a hospital prevalence figure for pressure ulcers of 10.2% (categories 1–4) [23]. The NHS Safety Thermometer has recorded an overall prevalence of 7.4% (categories 2–4) across all settings. The differences can be explained by the exclusion of category one pressure ulcers and provide some assurance that aggregated data from the NHS ST is similar to known prevalence from research.

### Caution is required in interpretation of these preliminary data

Comparisons between organizations or teams are not recommended since variations in interpretation of operational definitions, data collection systems, skills and case mix lead to variation between locations. However, for transparency, data are presented to help organizations compare their performance. To ensure the tool is robust enough for comparison in the future, training tools are being developed to help users apply the operational definitions consistently. Moreover, commissioners are being trained to assess the quality of data collection on site and work with the organizations to improve.

### Modifications to the sampling method can deliver efficiencies

Throughout testing we adhered to design principles for the instrument development. A primary aim was to ensure that the instrument could be completed in <10 min per patient and carry out the survey on ‘just enough’ patients. Initially, we asked for 50% of patients to be surveyed, this had to be increased to 100% in order for us to use statistical process control charts to measure progress which added additional burden. However, our survey data demonstrated that over 70% of the responding participants were able to complete the instrument in the allocated time. Reports from the site coordinators collected at feedback indicated that the time taken to collect data reduced with familiarity with the method and operational definitions to 5 min or less. Using a monthly sample ensured that the data collection burden was minimized. Initial concerns about the burden of data collection reduced considerably once users began to use the instrument, with many reporting stories of immediate action to improve care following data collection. Examples of how the NHS ST has brought about change in practice can be seen in case studies from 2012/13 CQUIN Guidance [24].

### Co-production by local organizations working with a national organization is helpful

Our theory that engagement in PDSA testing would lead to buy in and ownership was shown to be true in part, but also produced some unexpected and potentially interesting results. Frustration was expressed at the constant changing nature of the instrument by a small cohort of testers and a desire for it to be ‘finished’ intensified over time, suggesting some dissatisfaction with the iterative and unstable nature of the approach. Theories of the diffusion of innovation may help to explain this phenomenon in suggesting that over 50% of the hospital staff population would prefer a ‘tested’ product [25].

### Not all issues can be resolved through co-production

In a small number of cases, the testing was unable to produce an agreed outcome despite multiple iterative cycles. An example of this was the development of two operational definitions where there was an absence of consensus on the final outcome measures. In both cases (catheter acquired UTI and VTE), the outcome measures adopted in the NHS ST are surrogates for actual outcome measures because it was impossible to develop an outcome agreeable to experts within the design principles of the instrument. The notion of adopting an imperfect but stable operational definition is understood by improvement scientists but is less familiar and acceptable to content experts. Our experience suggests that a shared understanding of operational definitions, which can be used to track change over time, is critical to progress. Despite this, at the time of publication, there are ongoing concerns from content experts that data from the NHS ST could be used for judgement (performance measurement) rather than improvement and that operational definitions will be misinterpreted.

## Conclusion

It is possible to obtain national data through aggregation of data collected at the point of care. Co-production is helpful but cannot resolve all issues. Some caution is required in interpreting data and work is required locally to ensure data collection systems are robust and data collectors trained in the method. Sampling is an important strategy to optimize efficiency and reduce the burden of measurement. At the time of writing, July 2013, the NHS ST is being used by over 700 healthcare providers, including community settings and nursing homes. The overall impact of establishing this national measure has been reported in the ‘Safety Express’ case study and work continues to scale-up the improvement activity across England [26].

## Funding

The development of this work was funded by the Department of Health through the QIPP Safe Care workstream, supported by NHS England. Funding to pay the Open Access publication charges for this article was provided by Salford Royal NHS Foundation Trust.
